# Supplementary material for: Are carbon emissions trading and green financial instruments synergistic? -Comprehensive quantitative research based on content analysis
Source: PLoS One. 2024 Mar 7;19(3):e0298601. doi: 10.1371/journal.pone.0298601 (PMC10919601; doi:10.1371/journal.pone.0298601)
Supplement: S1 Data — (ZIP) [file pone.0298601.s001.zip › Data/地级市Z_F工作报告.zip!/║╙─╧/╓▄┐┌╩╨/└┤╫╘╣1⁄2╓┌║┼í╛┬φ┐╦╩2╛▌═°í┐_2012.docx]

各位代表：

　　现在，我代表市人民政府，向大会报告政府工作，请予审议，并请各位政协委员和其他列席人士提出意见。

　　一、过去五年的工作

　　过去的五年，是周口发展进程中极不平凡的五年。五年来，在省委、省政府和市委的正确领导下，市人民政府团结带领全市人民，深入贯彻落实科学发展观，积极应对各种困难和挑战，坚定不移抓发展，脚踏实地促转型，扎扎实实惠民生，圆满完成了二届人大历次会议确定的各项目标任务，全面开创了周口经济社会发展的新局面。

　　国民经济健康发展，综合实力持续攀升。五年来，我们牢牢把握发展第一要务，坚持“四个重在”的实践要领，围绕做大经济总量、推动产业升级、夯实发展基础、关注改善民生的发展要求，创新体制机制，突出重点工作，国民经济保持了快速持续健康发展的良好势头。2011年，全市生产总值1414.7亿元，是2006年的1.7倍；人均生产总值是2006年的1.8倍，五年年均增长12.5%，高于全省平均水平，初步遏制了与全省差距不断拉大的趋势。财政一般预算收入48.7亿元，是2006年的3.2倍；财政一般预算支出244.1亿元，是2006年的3.1倍；经济建设、改善民生、行政运转等有了一定的运作空间和回旋余地。全社会固定资产投资851.8亿元，是2006年的3.4倍；一些大型基础设施相继建成，生产生活环境明显改善。城镇居民人均可支配收入14583.5元，农民人均纯收入5447.6元，分别是2006年的2倍和2.1倍；城乡居民储蓄存款余额906.7亿元，是2006年的2.1倍。

　　经济结构日趋合理，产业层次持续提升。2011年，二、三产业比重达到72.4%，较2006年提高5.6个百分点。工业主导地位更加突出。培育壮大工业经济，着力打造产业集聚区，强力推进食品加工、纺织服装、医药化工三大支柱产业发展，工业对国民经济的支撑作用进一步增强。2011年，规模以上工业增加值465.1亿元，是2006年的2.6倍；主营业务收入1886亿元，是2006年的4.6倍；工业对全市生产总值的贡献率达42.2%。规模以上工业企业达到969家，益海粮油、财鑫集团等9家企业跻身全省“百强百高”企业行列，龙头企业的带动作用更加突显。农业基础地位不断加强。严格耕地保护制度，落实强农惠农政策，推进农业结构调整，扎实开展“两区一体系”建设。2011年粮食总产达到149.4亿斤，较2006年增产22亿斤，连续八年丰产增收。农民专业合作社发展到3788家，推动土地流转152.6万亩，发展大中型规模养殖场1625个，肉、蛋、奶产量分别较2006年增长11.1%、20.4%和112.3%。积极推进林业生态建设和林产业发展，10个县市区全部通过林业生态县验收。服务业得到长足发展。拓展服务业发展领域，改造提升传统服务业，大力发展现代服务业，努力增加服务业总量。新建改建黄淮物流港农产品批发市场等大型商业网点，万果园、一峰等超市逐步向规模化、集团化方向发展。2011年社会消费品零售总额579.8亿元，是2006年的2.5倍。旅游总收入58.8亿元，是2006年的2.5倍。非公有制经济快速发展。加强政策扶持，优化发展环境，着力推动非公有制经济健康快速发展。全市累计新增私营企业1.1万家、个体工商户10万多户。非公有制经济占GDP比重达到61.6%，较2006年提高5.3个百分点。节能减排扎实推进。建立健全长效机制，大力淘汰落后产能，积极推进环境综合整治，节能减排完成省定目标任务，环境质量持续改善。

　　项目建设加快实施，发展后劲持续集聚。坚持把项目建设作为引领经济社会发展的突破口，千方百计积极争取上级资金和项目支持，每年筛选100个重点大项目，并从中选出十五项重点工业项目和十五项基础设施项目，列为“双十五工程”，催生了一批新的经济增长点。五年来，共实施项目560个，完成投资497亿元，项目数量和质量均实现大的飞跃，综合带动作用更加突显。坚持“三规合一”、“四集一转”、产城融合，完善规划布局，加强扶持引导，开展观摩点评，推动产业集聚区建设上规模、上水平。全市产业集聚区建成区面积达到83.8平方公里，累计投资超过800亿元。2011年，产业集聚区规模以上工业主营业务收入953亿元、从业人员11.6万人，分别占全市规模以上工业的50.5%和46%。一些产业集聚区主导产业突出，发展特色鲜明，已成为转型升级的突破口、招商引资的主平台、吸纳就业的主渠道和县域经济发展的增长极。

　　基础设施不断完善，城乡面貌持续改观。适时调整投资方向，着力加快交通、能源、水利、城市基础设施等重点项目建设。五年全社会固定资产投资3290.9亿元，是上一个五年的3.7倍。综合交通枢纽建设成效显著。大广、南洛、周商、永登四条高速公路（周口段）全线通车，新增高速公路通车里程118公里，改善国、省干线公路876公里，提升农村等级公路6800公里，公路通车总里程达2.4万多公里，位居全省第4位，路网结构更加完善，被国家规划为公路运输枢纽城市。漯阜铁路（周口段）电气化改造即将竣工，沙颍河周口以下航道实现常年通航。我市在全省率先构建了公（路）铁（路）水（路）一体化综合交通运输体系。能源、水利建设取得较大进展。生物柴油年生产能力达到10万吨，年生物质发电量3亿多千瓦时，年产沼气1亿多立方米，年供应天然气5700万立方米，新能源开发利用初具规模。首座500千伏变电站建成投运，城乡配网不断完善，全市实现户户通电。沙颍河、涡河近期治理工程顺利完成，防洪标准达到20年一遇。新增有效灌溉面积205.8万亩。累计解决234.2万农村人口饮水不安全问题。城乡基础设施建设全面加强。中心城区和县城建设加快推进，八一路桥、体育场、周口大道、市文化艺术中心等一大批基础设施项目在建或投用。全市新增城镇建成区面积67.9平方公里、人口102.7万人，城镇化率达到31.5%，年均提高1.5个百分点，是我市历史上城镇化推进最快的时期。县市区全部建成垃圾和污水处理设施，84个建制镇垃圾中转站正常运行。扎实推进新型农村社区建设，44个新型农村社区部分已实现搬迁入住。全市农村实现了广播、电视全覆盖和村村通电话、乡乡通宽带，新农村建设迈出坚实步伐。

改革开放深入推进，经济活力持续增强。坚持深化改革、扩大开放，推进体制机制创新，经济社会发展活力进一步释放。重点领域改革扎实推进。全市151家国有工业企业基本完成改制任务，16家市属国有及国有参股企业改制工作有序推进。政府机构改革全面完成，医药卫生体制、文化体制改革加快推进，警务体制改革取得突破。

探索实施土地开发利用管理“三项机制”，开展农村土地整治和城乡建设用地增减挂钩，基本保障了发展用地。深化集体林权制度改革。扎实开展村民一事一议筹资筹劳工作。加快投融资体制改革，组建市综合投资公司、农业综合开发公司等5大投资公司，8家担保机构获得经营许可，国有控股银行不良贷款率大幅下降，市城市信用社成功改制为城市商业银行，并被国家银监会批准升级为周口银行。水利、教育、道路运输管理体制等改革不断深化。对外开放不断深入。创新招商引资理念和方式，主动承接产业转移，我市被省政府命名为“河南省制鞋产业出口基地”。加强与省直厅局和央企的合作，承办和自办“5 2”经济合作活动、周口—东莞制鞋产业合作交流洽谈会、中部八省非物质文化遗产展演等大型商贸文化活动，与129个国家和地区建立了经贸关系，雨润集团、晋煤集团等国内知名企业和益海粮油、富士康集团等世界500强企业相继落户周口。5年实际利用外资7.4亿美元，引进省外资金800.6亿元。企业自主创新能力不断提高。先后与河南工业大学、河南省科学院、河南省农科院等高等院校和科研院所建立科技合作关系，鼓励和引导企业加快创新平台建设，新建省级工程技术研究中心17家、国家级企业技术中心3家，建成了全国市级单位唯一锅炉能效测试中心。累计投入资金183.7亿元，实施科信电缆碳纤维导线等240个技术改造项目，远东生物“除草剂安全添加剂”等3个产品被认定为国家重点新产品，恒昌计量、瑞特电气等4家企业被列入国家科技型中小企业创新基金项目。年专利申请量突破600件。拥有中国驰名商标10件，省著名商标83件，省知名商标28件。莲花股份成为全省唯一首届“国家商标战略实施示范企业”，金丹乳酸公司荣获省长质量奖和国家科技进步二等奖。

　　和谐社会逐步构建，民生民计持续改善。着力办好事关民生的“十大实事”和“十项重点民生工程”，统筹发展各项社会事业，和谐周口建设迈出新步伐。民生改善成效显著。用于改善民生的支出逐年增长，连续4年占财政总支出的比重超过50%，2011年达到72.9%。就业规模持续扩大，累计新增城镇就业人员40.5万人、农村劳动力转移就业65万人，我市被列为创建省级创业型城市试点。医疗卫生、社会保障体系不断健全，并向农村延伸，基本实现应保尽保。调整提高了最低工资标准。建立社会救助和保障标准与物价上涨挂钩联动机制，有效保障了低收入群体的基本生活。加快保障性住房建设，新增保障性住房274.1万平方米，改造棚户区52万平方米，困难群体住房难问题得到缓解。大力发展慈善事业和残疾人事业，募集资金1.27亿元用于扶贫济困。各项社会事业全面进步。科普工作得到加强，8个县市被命名为全国科普示范县市。制定实施教育、人才中长期改革发展规划，建立完善教育投入保障机制和教师补充长效机制，贫困家庭学生资助实现从小学到大学全覆盖；全力推进中小学布局调整和校舍安全工程，投入资金8亿多元，新建、改建、扩建各级各类学校、幼儿园1500多所，市委党校新校区、周口卫校新校区、周口幼师新校区、第一实验小学等建成投用，周口职业教育园区被省政府确定为省重点建设示范性职业教育园区，周口科技职业学院被教育部认定为“国家级重点示范学校”，14所高中成为“省级示范性学校”，高招成绩连续5年位居全省前列。积极开展全民健身活动，竞技体育水平不断提升。实施文化精品工程，繁荣文化艺术事业，“周口文化现象”叫响全省，《都市长虹》、《云锦人家》、《老子》、《天职》等一批文艺作品荣获国家和省级大奖，“周末一元剧场”荣获国家社会文化活动政府最高奖“群星奖”。中国作家协会确定我市为文学创作基地。加强重大疾病防治和卫生应急工作，加快中医药事业发展，持续抓好“五大工程”建设，医疗卫生服务体系逐步健全。完善计划生育利益导向机制，稳定保持低生育水平。社会保持和谐稳定。不断加强和创新社会管理，有效解决影响社会和谐稳定的突出问题，及时化解人民内部矛盾，妥善处置各类突发事件，集中开展食品、药品安全专项整治，严厉打击违法犯罪，安全生产形势持续稳定好转，平安周口建设成效明显。

　　民主法制建设有力，精神文明持续加强。全面推进依法行政和法治政府建设，出台和完善了26个法治政府建设方面的文件，“五五”普法顺利完成，“六五”普法启动实施。自觉接受人大及其常委会监督，支持政协履行职能，认真办理人大代表建议和政协提案。密切联系工会、共青团、妇联等人民团体，广泛听取社会各界意见和建议。廉政建设和反腐败斗争取得新成效。深入开展民族团结进步创建活动，民族宗教工作得到进一步加强。开展不同形式的文明创建实践教育活动，大力弘扬新时期“三平”精神，公民思想道德素质和社会文明程度不断提升，涌现了杨正超、李灵、王文田、谢凤运、李高峰等一批在全国有影响的先进典型，树立了周口良好对外形象。大力支持军队建设，积极推进军民融合式发展，驻周人民解放军和武警部队在支持地方建设、维护社会稳定等方面作出了重要贡献，军政军民团结进一步巩固。外事、侨务、对台工作进一步加强，统计、审计、海关、新闻出版、史志、气象、地震、人防等工作取得新成绩。

　　各位代表!5年来之所以取得这些成绩，是省委、省政府和市委正确领导的结果，是市人大、市政协监督支持的结果，凝聚着全市上下同心同德、克难攻坚、锐意进取的心血汗水，铭刻着周口人民与时俱进、干事创业、奋发有为的壮志豪情，承载着一直关心支持周口发展的老领导、老同志和各界朋友们的深情厚谊。在此，我代表市人民政府，向全市广大工人、农民、知识分子、干部、驻周部队指战员、武警官兵、公安干警以及各界人士，致以崇高的敬意！向中央、省驻周单位、在周口创业发展的各位投资者和建设者，以及所有关心支持周口现代化建设的同志们、朋友们，表示衷心的感谢！

　　各位代表!过去的5年，是我市抢抓机遇、应对挑战、追赶跨越的5年，是全市上下敢于担当、开拓创新、奋发有为的5年，是实现富民强市迈出坚实步伐的5年。5年来，我们积极探索并走出一条符合周口实际的发展路子，为今后的发展积累了经验，主要有：坚持把实现追赶跨越、富民强市作为根本任务。着眼于全国、全省大局谋求自身发展，把贯彻科学发展观与立足周口实际结合起来，明确提出“加快三个转变”和“三个推进”的发展思路，遵循规律，有效运作，努力使工作不断有新突破、实践不断有新创造、发展不断有新跨越。坚持把推动“三化”协调科学发展作为总体要求。着眼于“三化”互为支撑、三次产业融合发展，突出新型城镇化的引领作用，发挥新型工业化的主导作用，巩固新型农业现代化的基础作用，依托优势，扬长避短，持续探索传统农区实现“三化”协调科学发展的新路子。坚持把持续推进改革开放作为根本途径。着眼于国家宏观战略的调整和地区产业加快转移孕育的发展机遇，把激活内力与借助外力结合起来，深入推进改革开放，在政策调整中抢占先机，在承接转移中赢得主动，不断化解各种矛盾和瓶颈约束，努力增创发展新优势。坚持把项目带动作为重要抓手。准确把握周口市经济社会发展的阶段性特征，把整体推进和重点突破有机结合起来，大力实施项目带动，立足当前，兼顾长远，努力推动一批投资规模大、带动作用强的重大项目落地建设。坚持把保障改善民生作为最终目的。立足于人民群众美好期待和市域财力相对不足的现实，把加快发展与改善民生有机统一起来，切实解决好人民群众最关心、最直接、最现实的利益问题，使改革发展成果惠及广大人民群众，努力在加快发展中改善民生、在改善民生中促进和谐发展。坚持把真抓实干作为为政之基。按照“三具两基一抓手”、“两转两提”要求，把领导方式转变与发展方式转变结合起来，统一思想认识，转变工作作风，提高行政效能，推动务实发展，在全市上下着力营造心无旁骛抓发展、心齐风正重实干的浓厚氛围。

　　各位代表！在看到成绩的同时，我们也清醒地认识到，人口多、底子薄、基础弱、经济总量小、人均水平低的基本市情还没有根本改变；经济增长方式粗放，产业结构不合理，转变发展方式任务艰巨；破解“钱从哪里来、人往哪里去、粮食怎么保、民生怎么办”4道难题，困难依然很大。在经济发展外部不确定因素增多、资源环境约束加大、区域竞争日趋激烈的大背景下，保持经济平稳较快发展，缩小与全省平均水平的差距，难度明显增大；中心城市发展相对滞后，辐射带动作用不强;项目总量不足、质量不高，大项目严重匮乏；社会事业发展历史欠账较多，改善民生、维护稳定的任务繁重；政府自身建设还存在不少薄弱环节，一些部门职能转变还不到位，行政服务效能不高，一些干部素质能力、运作水平还有待提升，少数干部以权谋私、奢侈浪费、消极腐败等现象仍然存在。对此，我们必须高度重视，时刻保持清醒的忧患意识，采取有效措施，认真加以解决。

　　二、今后五年工作安排

　　各位代表，尽管我们前进的道路上还存在不少困难和问题，也可能出现新的矛盾和挑战，但总体来看，今后5年是周口全面建设小康社会的关键时期，是加快转变发展方式的攻坚时期，也是我们必须紧紧抓住并可以大有作为的重要战略机遇期。从外部环境看，经济全球化深入发展，国际和沿海地区产业转移步伐加快，有利于借助外力、提升发展层次；国家实施扩大内需战略，持续加大对“三农”、民生改善、社会事业的投入力度，有利于争取政策支持，筹集建设资金；国家促进中部崛起、中原经济区建设全面推进，有利于促进“三化”协调发展，提高区域竞争力。从我们自身看，全市加快发展的内生机制初步形成，探索出了符合周口实际的发展思路，富民强市的愿望更加强烈，干事创业的氛围更加浓厚。我们必须抢抓难得机遇，积极应对挑战，坚定信心，科学决策，克难攻坚，不断开创跨越发展的新局面。

　　今后5年，按照市三次党代会确立的发展思路，政府工作的总体要求是：深入贯彻落实科学发展观，以科学发展为主题，以加快转变经济发展方式为主线，以富民强市为目标，坚持“四个重在”实践要领，紧紧抓住建设中原经济区的重大机遇，着力实施项目带动，着力开展招商引资，着力推进产业集聚，着力保障改善民生，全面增强综合实力、发展活力和区域竞争力，持续探索走好一条不以牺牲农业和粮食、生态和环境为代价的“三化”协调科学发展的路子，促进经济社会又好又快发展和社会和谐稳定，为全面建设小康社会打下坚实基础。

　　今后5年的奋斗目标和主要任务是：

　　——综合实力显著增强。围绕把周口打造成为中原经济区豫东南区域增长极，全面培育现代农业示范区、承接产业转移重点区，着力建设农产品精深加工产业中心、豫东南商贸物流产业中心、人力资源开发中心 “一极两区三中心”的战略定位，聚精会神搞建设，一心一意谋发展，持续保持高于全省的发展速度，大幅缩小与全省平均发展水平的差距，力争到2016年生产总值突破2400亿元，公共财政预算收入突破100亿元，城镇化水平、工业整体实力、城乡公共服务均等化水平快速提升，在中原经济区建设中的战略支撑明显增强，全省发展有地位，区域发展有影响。

　　——经济结构更趋合理。强力实施项目带动、开放带动战略，加快产业集聚区建设，构建现代产业体系、自主创新体系。夯实农业基础地位，稳定提高粮食生产能力，2016年全市粮食生产能力达到165亿斤。实施工业倍增计划，工业增加值突破1400亿元，规模以上工业增加值突破1000亿元，建成在全省乃至全国有一定影响的食品加工、纺织服装、医药化工、制鞋加工基地。实施“巨人成长”工程，培育10家年销售收入50亿元、5家年销售收入100亿元以上的企业或企业集团。发展壮大战略支撑产业，加快发展培育新兴产业，做大做强基础产业，强力推进现代服务业，二、三产业比重达到80%以上，科技进步对经济发展的贡献率达到52%以上。积极发展循环经济、低碳经济，促进经济绿色增长，能源资源利用效率明显提升，万元GDP能耗进一步下降，主要污染物排放量有效控制，生态环境质量明显改善，经济发展与人口、资源、环境更加协调。

　　——城乡社会一体发展。新型城镇化引领作用明显提高，城乡一体化进程明显加快，基本形成“一中心、五组团、五支撑”的发展格局，城镇化率超过40%，中心城区建成区面积达到100平方公里，人口达到100万人。城乡基础设施更加完善，一些打基础、管长远、增后劲的基础设施项目相继建成；城镇规划建设和管理水平明显提高，“脏乱差”得到全面治理，城乡面貌焕然一新。中心城市的辐射带动作用明显增强，县域城区和中心城镇更具特色，新型农村社区建设加快推进。文化建设融入城市血脉，宜居、宜业、宜游的城市特色更加鲜明，现代城镇体系和现代产业体系基本形成。

　　——基础设施更加完善。持续加快高等级公路建设，建成周口至新郑机场、周口至南阳的高速公路，建设中心城区至项城、淮阳、西华快速通道，实现商水与中心城区南北主干道路的对接，完善国家公路枢纽城市功能；完成漯阜铁路周口段改造，高标准建成周口新火车站和汽车综合客运总站，增加客运车次和直达城市数量；提高沙颍河通航标准，加快周口港口物流产业集聚区项目建设，实现港口经营规模化；建设周口通用机场，在全省率先构建公（路）铁（路）水（运）空（航空）一体化综合交通运输体系，形成新的独特优势。建成2×30万等级燃气热电厂和豫东煤炭储配基地，力争建成周口2×60万千瓦燃煤电厂、周口平原水库等项目，城乡基础设施体系更加完善，要素保障能力显著提升，综合竞争能力明显增强。

　　——改革开放深入推进。坚持社会主义市场经济改革方向，充分利用国务院《指导意见》赋予的先行先试权，以更大的决心和气魄深化体制改革，加强机制创新，破解发展难题，力争在重点领域和关键环节取得新突破，加快构建有利于科学发展的体制机制，全社会的创新创业创造活力逐步增强。不断创新资金、土地、人力资源、能源电力等要素配置机制，为经济社会发展提供坚强保障。充分发挥承接东部产业转移的前锋作用，全方位实施开放带动战略，积极承接产业转移，提高利用外资水平。建成海关特殊监管区，为更高层次对外开放搭建平台，促进开放型经济快速发展，进出口总额和实际利用外资实现翻一番，增强在淮海经济协作区乃至全国的影响力。

　　——社会发展再上台阶。覆盖城乡居民的社会保障体系逐步完善，力争到2016年，全市城镇职工和居民医保参保率达到90%以上，新农合参保率稳定在95%以上，城镇登记失业率控制在4.2%以内，城镇居民人均可支配收入达到2.2万元，农民人均纯收入达到8300元，全市人民各项社会保险政府补助标准、干部职工工资标准跨入全省中等以上水平。住房公积金覆盖率达到全省平均水平。累计新建和改扩建标准化中小学校200所、公办幼儿园200所，实现每个乡镇最少有1所标准化幼儿园。每千人拥有执业医师数、注册护士数、病床数赶上全省平均水平。人口自然增长率低于全省平均水平。基本公共服务均等化程度提升，公民思想道德素质、科学文化素质和健康素质大幅提高。民主法制和精神文明建设进一步加强，社会大局保持和谐稳定。

　　各位代表！“合抱之木，始于毫末；九层之台，起于垒土”。发展需累日之功，跨越非一蹴而就。实现克难攻坚、跨越发展，争先进位、富民强市，是1000多万周口人民的共同事业，我们必须放眼长远、立足当前，团结一心、合力攻坚，以时不我待的紧迫感、跨越发展的使命感、勇于担当的责任感，从现在做起，从点滴做起，在迎接挑战中把握机遇，在服务大局中谋划发展，创造持续跨越的周口速度，书写富民强市的崭新篇章。我们坚信，经过5年或更长一些时间的努力，一个走上复兴之路、富民之路、幸福之路的新周口，必将再现“万家灯火侔江浦，千帆云集似汉皋”的历史辉煌！一个更有实力、更具活力、更富魅力的新周口，必将焕发出更加璀璨夺目的光彩！

　　三、2012年主要工作

　　根据市三次党代会和市委经济工作会议精神，今年经济社会发展主要预期目标是：生产总值增长11%左右；公共财政预算收入增长12%；全社会固定资产投资增长21%以上；社会消费品零售总额增长16%；实际利用外商直接投资增长25%；外贸出口总额增长22%；城镇居民人均可支配收入、农民人均纯收入均实际增长9%；城镇新增就业7万人；人口自然增长率控制在6.5％。以内；居民消费价格涨幅为4%左右；节能减排完成省定目标。

　　围绕上述目标，做好今年的各项工作，必须把握“抢抓机遇、持续求进、又好又快”的工作总基调，从以下八个方面加压加力，奋力突破：

　　（一）大力实施项目带动战略。以项目建设扩大投资规模、调优经济结构、增强发展后劲、提升区域竞争力。

　　抓好重点项目建设。今年全市拟定138个重点项目，总投资522.7亿元，年度计划投资206.3亿元，并从中筛选出十五项重点工业项目和十五项重点基础设施项目，继续实施“双十五工程”。围绕这些项目，在土地、资金担保、环境容量指标上给予倾斜，努力提高项目资金到位率、开工建设率和建成投产率。

　　提高项目运作水平。进一步健全完善项目联席会议、项目建设进度月通报等制度，继续采取领导分包、现场办公、观摩点评、督查督导等办法，按照“新上项目抓开工，在建项目抓进度，建成项目抓运营”的要求，着力解决项目谋划跟不上、前期不扎实、推进抓不紧等问题，确保项目建设顺利推进。对 “双十五工程”项目，坚持市、县领导双重分包和责任单位、项目所在地政府、业主单位三级协调机制，分层级、分战线合力推进。进一步落实项目建设主体责任，强化监督，跟踪问效，严格奖惩。

　　扎实做好项目储备。深入研究国家产业政策，准确把握国家宏观调控的方向、力度和节奏，加大向上争取项目资金的力度。结合我市产业基础、资源禀赋、社会发展需要，重点在“三农”、民生、交通能源、产业结构调整、节能减排、自主创新、社会事业等领域，谋划一批事关经济社会发展全局、技术含量高、经济效益好、带动能力强的大项目，争取有更多的项目进入国家、省扶持的范畴。

　　（二）持续扩大对外开放。坚持把对外开放作为应对复杂局面和破解各种制约最直接、最有效、最综合的战略举措，积极实施开放带动主战略，大力承接产业转移，构建全方位、宽领域、多层次的开放格局，努力以开放促发展、促改革、促创新、促转变、惠民生。今年争取利用外商直接投资完成3.6亿美元，利用省外资金307亿元。

　　扩大对外开放。开展“承接产业转移突破年”活动，力争全年签订承接省外产业转移项目100个以上、产业转移资金200亿元以上。加快服务业对外开放，吸引境内外投资者进入现代物流、金融、旅游、文化等领域。深化农业对外开放，重点引进品种培育、精深加工等高技术项目以及畜牧、果蔬等高效农业项目。扩大基础设施和城乡建设领域对外开放，鼓励各类资本参与公路、水利、城市建设等基础设施建设运营。注重发挥大型招商活动的平台作用，认真组织参加第十六届中国国际投资贸易洽谈会等国家、省举办的大型招商活动，有针对性自主举办周口（广东）制鞋产业合作交流洽谈会、周口（浙江)纺织服装产业合作交流会、“百名鞋商周口行”活动等大型招商洽谈会，着重吸引和承接“两头在外”的台、粤、闽、浙等地制鞋、制衣产业。下大力气抓好与深圳大型物流项目洽谈工作，促其早日落地。建立健全与央企、省直厅局合作长效机制，重点引进一批以机械制造、电子信息、新材料、新能源、生物医药为主的高新技术和机电类企业，改善优化我市产业结构，带动其他产业快速发展。

　　优化开放机制。树立开放“一盘棋”思想，健全开放领导体制，建立联动协作机制，形成各司其职、各尽其力、配合联动的举市开放长效机制。充分发挥各类商会、行业协会、中介组织联系面宽、贴近市场的优势，加强与珠三角、长三角地区的商会、行业协会等民间组织的联系，形成市场化的承接模式。加大招商引资绩效评估力度，严格落实《周口市承接产业转移工作考核办法》。优化开放环境，提升服务水平，重诺守信，营造亲商、安商良好氛围。开展“招商引资项目落实年”活动，强化签约项目落实，确保项目早建设、早投产、早见效。继续完善进出口重点企业联系制度，着力抓好莲花味精、金丹乳酸、宝昌鞋业等重点企业出口运行，保持出口持续稳定增长。加快“河南省制鞋产业出口基地”建设，强化基础设施，健全配套政策，完善产业链条，尽快建成鞋业专业市场和物流配送中心，培育外贸出口新的增长点。

　　注重开放实效。着眼于产业优化升级，实现招商引资由注重规模数量向注重质量效益转变；着眼于“三化”协调发展，实现由注重工业领域招商向全方位拓展转变；着眼于服务水平提升，实现由主要靠政策优势招商向靠发展平台、发展环境招商转变，使自身开放发展水平与中原经济区“三化”协调科学发展相适应、相统一。

　　（三）全力推进新型城镇化。在统筹推进中心城市、县城、小城镇和新型农村社区建设的基础上，突出中心城区建设这个薄弱点和新型农村社区这个新课题，加快构建符合农区实际、具有周口特色的现代城镇体系。

　　做大做强做好中心城市。继续完善城市总体规划，科学制定周商项淮西组团式发展实施方案。按照完善配套设施、提升城市功能、聚集优质产业、强化精细管理的要求，推进川汇区、开发区、东新区对接融合发展。加大中心城区棚户区和城中村改造力度，实施6条老城区道路改造，加快八一路桥、新火车站广场等在建项目进度，做好东外环路、沙颍河中心城区段综合改造前期准备工作，年内开工建设市行政中心、周口市污泥处置厂、北环路改造等项目。坚持产城融合、产城互动。规划建设新的特色商务区（街），推动黄淮物流港农产品批发市场等项目建设。落实好《关于进一步加快中心城区工业发展的实施意见》，强化各类生产要素保障，整合资源，集聚发展合力。痛下决心，多措并举，切实改变重建设、轻管理倾向，加强和改进城市管理，理顺管理体制，逐步解决城市管理多头执法问题。启动中心城区创建国家级园林城、国家级双拥模范城和省级卫生城、省级文明城活动，调动广大市民参与城市管理的积极性和主动性。持续开展交通秩序、环境卫生、市容市貌、“两违”治理综合整治行动，改善城市形象，提升城市品位。

　　有序发展中小城镇。加强县城和9个中心镇建设，积极指导和帮助项城市高寺镇开展第三批全国发展改革试点镇建设工作。把县城和中心镇作为统筹城乡发展的重要支撑，发挥自身优势，强化产业培育，完善公共服务，不断提高县域城镇承接产业转移、承接中心城市辐射、带动农村发展的能力。放宽中小城市落户条件，尽快建立完善农民工城镇入户机制并形成制度，合理引导人口流向，让更多农村富余劳动力就近转移就业。研究建立以农民变市民为核心的新型城镇化工作考核评价体系，逐步将城镇基本公共服务覆盖到农民工，切实解决农民进城就业、户籍、住房、社会保障、子女入园入学等问题，使符合条件的进城农民真正转为城镇居民。

　　统筹发展新型农村社区。抓住国家加大对“三农”投入、中原经济区建设和东部沿海产业转移的难得机遇，按照“分类指导、科学规划、产业先行、就业为本、群众自愿、量力而行”的原则，创新思路，整合资源，集中财力、借助外力、调动民力，促进产业集聚区、现代农业示范区和新型农村社区联动推进，力求探索出一条贫困农业地区城乡一体、持续健康发展的新路子。做好新型农村社区布点和建设规划，启动20个新型农村社区建设。完善社区建设扶持政策，抓好典型带动，市、县都要选择建设试点，以点带面，稳妥推进。构建新型农村社区服务新体系，创新社会管理，引导农民转变生产生活方式。

　　（四）加速推进新型工业化。把推进产业产品结构调整作为加快发展方式转变、强化“三化”协调发展支撑的战略任务，加大政策支持和资金投入，突出抓好产业集群、载体建设、人力保障等关键要素和重点环节，做大总量，做优结构，不断提高周口工业发展的层次和水平。

　　壮大支柱产业。加大技术突破、链条延伸和改造升级力度，强化上下游企业合作，做大做强三大支柱产业。食品加工业以发展高附加值的品牌食品为重点，打造农副产品精深加工产业中心。鼓励五得利集团、金丝猴集团等企业，大力发展专用面粉、烘焙食品等面制品和休闲食品，拉长粮食精深加工链条。依托志元集团、雨润集团、六和集团、陈州华英、大用邦杰等企业，大力发展冷鲜分割肉、调理肉制品、熟肉制品等三大类主导产品。支持益海等骨干企业扩大生产规模。以宋河、四五等知名品牌为重点，扩大优势中高端酒类生产规模。纺织服装业重点推动产业升级和品牌建设，积极引进壮大符合环保要求、工艺水平较高、市场竞争力较强的印染企业和服装企业。依托扶沟、太康、项城专业园区，积极发展休闲面料等特色品种。依托淮阳、郸城专业园区，丰富服装产品结构，加强市场开拓。医药化工业以四方药业、辅仁药业、百年康鑫等企业为骨干，进一步增强自主研发能力，加快新型药品的研制，打造知名品牌。重点发展以金丹乳酸、财鑫糖业为龙头的生物化工，以晋鑫、昕州、绿原为龙头的化肥化工，以所望化妆为龙头的日用化工，推动化工行业企业可持续发展。同时，以科信电缆、银丰塑料、龙源纸业等企业为重点，加快发展通信电缆、生物科技、彩印包装等产业，着力培育产业发展新优势。

　　夯实产业载体。加快产业集聚区对外连接通道、环保、供电等基础设施建设，加强职工培训、物流仓储、检验检测、廉租房和公租房等服务设施建设，为进城务工人员提供生活便利，全面提升综合配套能力。综合运用项目准入、要素配置、利益分成等手段，强化主导产业集群培育，提升产业集聚效应。抓好9大行业48个专业园区建设，提升产业的高关联度、高附加值、高技术含量，把专业园区打造成市县区经济社会发展的增长极。今年每个产业集聚区都要有2～3个亿元以上项目落地，11个产业集聚区要完成各类投资200亿元以上。

　　培育核心优势。结合我市产业发展实际，围绕食品加工、纺织服装、医药化工、机械制造等重点领域的关键技术难题，集中优势科技资源，组织实施重大科技专项。加快自主创新体系建设，支持省内外科研机构、高校、大企业集团在产业集聚区设立研发中心、孵化器、检验检测服务平台，推动高新技术产业规模化。积极采用先进适用技术改造提升传统优势产业，组织实施80个重点技术改造项目。实施品牌带动战略，争创河南名牌6个以上，力争年内省著名商标达到90件、中国驰名商标达到10件以上。推进转型升级。以龙头带动、基地支撑、高端突破为着力点，大力发展电子信息、装备制造、新型建材等高成长性产业。以节能降耗、重组整合为着力点，培育壮大生物、新能源、新材料、高端装备等先导产业。加快信息化与工业化深度融合，实现技术进步和产业提升，打造数字企业。着力培育“专、精、特、新”中小企业，深入推进企业兼并重组，壮大一批拥有知名品牌和核心竞争力的大型企业集团。扩大融资渠道，积极筛选培育一批上市企业，推动金丹乳酸公司、新帅克药业、金丝猴集团、普爱饲料等企业尽快成功上市，引导企业由间接融资向直接融资转变。

　　积极发展服务业。坚持服务业优先发展理念，加大政策扶持力度，支持服务业关键领域、薄弱环节发展和品牌建设，提升综合服务功能。适应新型工业化和居民消费结构升级的新形势，加快发展信息服务、金融服务、科技服务、商务服务等生产性服务业，大力发展社区服务、家政服务、医疗卫生、休闲娱乐等生活性服务业。积极实施《现代物流业发展规划》，加快重点物流项目建设步伐，建设区域物流中心。在北京等大城市建设周口特色农产品展销大厅，构建周口农产品市场网络和展示平台。抓好农家店配送和配送中心建设，实现农资农家店乡镇全覆盖。

　　开发人力资源。围绕就业增收、招商引资和产业集聚区用工需求，实施职教攻坚和全民技能振兴工程。加快市职业教育园区、县职业教育中心、产业集聚区培训基地建设，大规模开展订单式培训，使广大务工者由低端劳动力资源转变为具有一定专业技能的人力资源。抓住市外务工人员回流增多机会，加大技能培训力度，提高再就业层次和能力。规范企业用工行为，创新企业用工模式，引导和支持企业下乡进村入户，促进更多农民工在家门口就业。

　　（五）巩固提升新型农业现代化。始终把“三农”工作摆在重中之重的位置，持续加强“两区一体系”建设，坚持保粮与增效并重，把建设现代化高标准粮田和发展现代农业产业化集群作为推进新型农业现代化的具体抓手，扎实推进国家重要的粮食生产和现代农业基地建设，加快农业大市向农业强市转变。

　　打造粮食生产核心示范区。大力实施高标准粮田“百千万”建设工程，年内每个县市完成5个万亩方、50个千亩方、500个百亩方高标准粮田建设任务。积极开展高产创建活动，推进以乡镇为单位的整建制粮食高产创建工作，抓好中低产田改造、土地整理、新增千亿斤粮食工程等项目。搞好灌区配套改造、小型农田水利和节水农业建设，大规模建设旱涝保收高标准基本农田，新增有效灌溉面积60万亩、旱涝保收田面积29万亩、除涝配套面积42万亩，夯实农业稳产增产基础，确保全年粮食总产达到150亿斤以上。严格保护耕地，确保耕地保有量不少于1280万亩。

　　建设现代农业示范区。围绕国务院《指导意见》“支持周口建设国家级现代农业示范区”的定位，科学谋划，大胆探索，加快建立“一县一品”、“一县一特色”的国家和省级现代农业示范区。大力实施“50个农业产业化生产基地建设工程”和“50家龙头企业培育工程”，打造农业产业集群，力争年内每个县市区各培育一个销售收入20亿元以上的农业产业化集群。大力发展良种繁育和种子产业，完成全国小麦育种创新基地建设规划方案，加快周口市现代农业研究开发基地建设。实施标准化规模养殖提升工程和示范创建活动，新改扩建标准化畜禽养殖场（小区）180个。

　　健全农业服务体系。大力推动农业科技创新，积极参与实施国家粮食丰产科技工程。加快新型农业技术推广体系建设，扩建完善9个县级农业技术推广服务中心，建设50个以上的区域农业技术服务站，加快以乡镇为单位的基层水利服务体系建设。加强农产品质量检测体系建设，重点扩建完善市级和8个县级农产品质量安全检测中心，建立健全乡镇或区域性动物疫病防控和畜产品质量安全监管体系。大力开展农业实用技术培训，培训农村劳动力10万人次。

　　推进农业改革创新。加快土地流转服务平台建设，探索建立土地流转保障机制，确保农民土地承包经营权规范流转。创新农村金融服务，支持农村信用社、农村小额贷款公司健康发展，鼓励各类金融机构增加对“三农”的信贷投放，扩大农业政策性保险。创新农业经营机制，大力发展农民专业合作、土地股份合作等新型农业合作组织，积极发展多种形式的村级集体经济。全市各类农民专业合作社争取突破5000家。持续加大对“三农”的投入力度。健全财政支农投入稳定增长机制，进一步建立健全强农惠农政策体系。加强农村水电路气等设施建设，解决80万农村群众饮水不安全问题，改造县乡公路230公里。按照国家新扶贫标准，加大集中连片困难地区扶贫开发力度，脱贫16万农村人口，完成整村推进村100个。

　　（六）持续推进文化建设。发挥文化资源优势，加快文化改革发展，打造华夏历史文明传承创新区重要板块，不断提升发展软实力，以文化力提升经济力、创造生产力。

　　提升文化发展的影响力。围绕“伏羲文化、老子文化、姓氏文化、农耕文化”四种文化资源，建设华夏历史文化传承创新区的核心区，羲皇故都、朝祖圣地，老子故里、道家圣地，万姓同根、根脉圣地“一区三圣地”。以周家口明清历史文化传承创新为核心，规划建设关帝庙文化休闲产业园区和沙颍河文化休闲风光带；以太昊陵、龙湖、淮阳古城为核心，规划建设淮阳文化旅游产业集聚区；以老子文化为核心，规划建设鹿邑老子文化传承创新示范区。规划建设市文化创意产业园、伏羲文化研究基地、老子文化研究基地，加强汝阳刘笔业、泥泥狗、张氏泥塑等开发利用，培育周口特色文化和文化产品。推动太昊伏羲陵5A级景区和关帝庙4A级景区创建工作，打造以吉鸿昌将军纪念馆为主的红色旅游品牌，提升龙都朝祖庙会、老子庙会、女娲庙会等传统庙会的规模与档次，完善袁世凯故居、叶氏庄园、华威民俗文化博物苑、中华槐园等景区的基础设施和服务设施，不断提升我市旅游的内涵品位和吸引力。

　　激发文化发展的内动力。加快推进文化企事业单位改革，组建周口日报报业集团，完成国有文艺院团改革，扎实推进市文化局（市新闻出版局）、市广播电视局合并组建工作，规范推进电台电视台合并，积极推进县级有线电视网络整合，推动淮阳县文化改革发展试验区取得突破性进展。深化文化市场综合执法改革。引导和鼓励社会力量参与公共文化服务，鼓励支持民间资本进入法律法规许可的文化产业领域。

　　增强文化发展的感染力。大力实施公民道德建设工程，提高全民文明素质。组织开展“学习雷锋、弘扬‘三平’精神”教育实践活动，加强和改进未成年人思想道德建设和“三理”教育。继续开展“三优三创”、“清洁家园行动”和文明村镇等群众性精神文明创建活动。继续提升文化基础设施功能，建设1797个农家书屋，完成85个乡镇综合文化站和20个城市社区文化中心的设备配备任务。大力实施文化惠民工程，不断提高公共文化服务水平。着力实施文化艺术精品工程和品牌战略，进一步树立“周末一元剧场”、中部八省非物质文化遗产展演等国内知名文化品牌。加大知识产权保护力度，深入开展“扫黄打非”工作。

　　（七）强力推进改革创新。以更大决心和勇气全面推进各领域改革创新，力争在一些重点领域和关键环节取得新突破。

　　加大改革创新力度。深化行政审批制度改革和投资体制改革。稳妥推进事业单位分类改革。建立和完善财政预算公开制度，积极推进国库集中收付、公共支出制度化精细化管理。支持税务部门深化改革，强化征管。继续深化农村综合改革和集体林权制度改革。深化医药卫生体制改革，将基本药物制度实施范围扩大到村卫生室。积极稳妥推进商贸、物资、交通、教育、水利等行业改革。支持金融部门改革，推动农村信用社达标升级和股权改造工作，支持符合条件的农村信用社组建农村商业银行。落实支持非公有制和中小企业发展的各项政策措施，鼓励民间资本进入市政、能源、教育、医疗等领域，促进非公有制经济规范健康有序发展。

　　创新要素保障机制。认真落实《关于支持金融业发展的意见》，大力发展金融产业，做大银行业，发展保险业，壮大地方资本市场。积极实施“引行入周”战略，吸引全国性股份制银行进驻周口。大力推动银企、银政合作，争取更多信贷指标和项目调增额度，引导更多社会资金投入实体经济。积极推进百家“小巨人”企业信贷培育活动，努力破解中小企业融资难问题。进一步加强政府投融资平台建设，提高信用级别，增强融资能力。创新土地开发利用管理机制。完善土地开发利用管理“三项机制”，加强土地开发利用管理。实施差别化土地政策，计划指标重点保障中心城区的重点项目和重要基础设施用地；建设用地增减挂钩指标和“三项整治”节余指标，集中用于县域城镇及产业集聚区。扎实开展农村土地整治，保障新型农村社区及非农业发展用地。探索开展城镇建设用地增加规模与吸纳农村人口进入城市定居规模挂钩、城市化地区建设用地增加规模与吸纳外来人口进入城市定居规模挂钩。继续创新鼎祥制衣有限公司用工模式，破解企业用工难题。

　　搞好环境综合治理。突出抓好工业、交通等重点领域节能减排，严格控制污染物排放总量，不断改善环境质量。严格实行能源消耗强度和总量双控制，落实好省千家企业节能低碳等八大节能专项行动计划，并逐步开展循环经济试点工作。抓好贾鲁河、沙颍河等流域的水污染防治，省政府考核的5个河流断面达标率在60%以上。建成投运周口市沙北污水处理厂、沈丘县沙南污水处理厂等4个城镇污水处理工程，开工建设郸城县第二污水处理厂等3个污水处理工程。深入开展“创建国土资源节约集约模范县（市）”活动，确保国土资源有效保护、有序开发。

　　（八）致力构建和谐社会。把改善民生放在更加突出的位置，大幅度增加财政民生投入，扎实办好“十项重点民生工程”，以民生改善促进和谐稳定。

　　在保民生中实现和谐。支持劳动密集型和小型微型企业发展，鼓励以创业带动就业，多渠道开发就业岗位。重点解决农村劳动力、城镇就业困难人员和退役人员就业问题。加快完善城乡社会保障体系。建立健全最低工资标准、职工工资、退休人员基本养老金、城乡低保及农村“五保户”供养标准的正常调整机制，落实机关事业单位工资和津贴补贴制度。继续增加企业退休人员基本养老金。实现城乡居民社会养老保险制度全覆盖。落实社会救助和保障标准与物价上涨联动挂钩机制，增加对农村寄宿制学校和高校食堂临时性价格补贴。巩固扩大基本医疗覆盖面，城镇居民医保和新农合补助标准提高到每人每年240元。强力推进保障性安居工程建设，新开工各类保障性住房2.6万套、竣工6500套。

　　在解民忧中促进和谐。加大教育投入，在中心城区和县城新建12所小学、10所初中、40所农村寄宿制标准化学校。加强学校安全管理和教师队伍建设，完善国家助学制度，逐步将中等职业教育免学费政策覆盖到所有农村学生，扩大普通高中家庭经济困难学生资助范围。支持周口师范学院、周口职业技术学院等高校建设，为地方经济社会发展提供智力支持。继续实施县级医院扩增计划，进一步完善公共卫生服务体系。广泛开展全民健身活动，组织办好市第二届运动会。继续稳定低生育水平，确保人口自然增长率低于全省平均水平。深化殡葬改革，加快城乡公益性殡葬设施建设。大力发展社会福利和慈善事业、老龄事业、残疾人事业。

　　在顺民意中体现和谐。加强和创新社会管理，扎实推进“六五”普法依法治理工作，健全群众权益维护机制，深入开展领导干部接访活动，加强和改进信访工作。加强社会矛盾隐患排查和风险评估，完善大调解工作体系，积极开展安全周口创建活动。落实和完善安全生产制度，严格食品药品和食盐安全监管，强化社会治安综合治理，严密防范、依法打击各种违法犯罪活动，保障人民群众生命财产安全，确保社会大局和谐稳定。

　　认真落实党的民族政策，深入推进民族团结进步事业，促进各民族共同团结进步、共同繁荣发展。持续加强国防教育和国防后备力量建设，支持驻周解放军、武警部队、预备役部队、民兵和人民防空建设，促进军民融合式发展。深入开展双拥共建，巩固发展军政军民团结。支持工会、共青团、妇联等人民团体发挥桥梁纽带作用。继续做好审计、统计、海关、气象、地震、外事、侨务等工作。

　　四、全面加强政府自身建设

　　复杂多变的经济形势、艰巨繁重的发展任务，对政府工作提出了新的更高要求。我们必须更加注重政府自身建设，更加注重提高政府管理效能和服务水平，使政府工作符合经济社会发展和市委“五个三”的工作要求，树立“学习做表率、工作上水平、群众信得过”的良好政府形象，建设人民满意的政府。

　　（一）加快服务型政府建设。深化行政管理体制改革，加快职能转变，积极为各类市场主体提供优质服务，创造良好发展环境。创新公共服务体制和服务方式，依法管理和规范社会组织，建立健全科学有效的利益协调机制、诉求表达机制、矛盾调处机制和权益保障机制，维护社会公平正义。加强应急管理体系建设，有效应对自然灾害、事故灾难、公共卫生和社会安全事件，提高危机管理和抗风险能力。大力推进政务公开，确保权力公开透明运行。保障人民群众的知情权、参与权和监督权。完善重大事项调查研究、集体决策、专家咨询、社会公示与听证、决策评估等制度，推进决策的科学化、民主化。进一步规范和减少行政审批，简化办事程序，完善限时办结、首问负责、服务承诺等制度，提高办事效率，更好地为基层、企业和公众服务。

　　（二）全面推进依法行政。认真落实监督法，严格依照法定权限和程序行使权力、履行职责，自觉把政府工作置于市委领导之下，接受人大及其常委会的法律监督、工作监督和政协的民主监督，严格执行人大及其常委会的决议、决定，广泛听取各民主党派、工商联和无党派人士的意见和建议。建立健全符合科学发展观要求的经济社会发展综合评价体系和考核指标体系，推行“四制”工作法，开展政府绩效评估，建立行政问责制度，不让老实人吃亏，不让投机钻营者得利。加强政府法制工作，强化行政执法监督，推进行政执法责任制，加强行政复议工作。成立周口仲裁委员会，完善经济纠纷解决渠道。全面贯彻落实中央关于党风廉政建设、反腐败斗争的决策部署以及国务院第五次廉政工作会议精神，按照市委统一安排，把反腐倡廉建设与政府各项业务工作和制度建设紧密结合起来，规范行使行政权力，加强审计监督、效能监察，做到有权必有责、用权受监督、违法要追究。在土地征用、房屋拆迁、企业改制、环境整治等方面，严格按法定程序办事，坚决纠正损害群众利益的行为。抓好政府工作人员的教育、监督和廉洁自律，加强对重点领域的监管，严肃查处各类违纪违法案件，创新反腐倡廉工作体制机制，深入开展党风廉政建设和反腐败斗争。

　　（三）切实加强政风建设。深入开展“作风转变暨环境创优”和“走基层、听民声、解难题、办实事”活动，进一步增强执行力和落实力，确保市委决策及各项工作落到实处。弘扬求真务实精神，大兴求真务实之风，真正做到亲民、务实、进取、廉洁。增强工作责任感和紧迫感，始终保持只争朝夕、奋发有为的精神状态，集中时间和精力，多做一些事关全局、谋划长远、夯实基础的工作，多开展一些深入实际的调查研究，多解决一些影响改革发展稳定的突出问题，多办一些为人民群众排忧解难的实事。坚持勤俭节约，严格控制“三公经费”，严格财政支出管理，严格控制修建楼堂馆所，严格公费出国出境管理，严格规范公务接待行为，真正把有限的资金和资源用在关键处。大力精简会议、文件，切实改进会风、文风，力戒形式主义和官僚主义。树立终身学习思想，不断更新知识，提高理论素养和业务能力，建设学习型机关。进一步提高公务员管理的法制化、规范化水平，努力建设一支政治坚定、业务精通、清正廉洁、作风优良的公务员队伍。

　　各位代表！

　　蓝图已经绘就，实干决定未来。时代赋予我们重任，人民寄予我们厚望。面对新的形势和任务，我们深感责任重大；展望周口未来，我们充满信心。让我们在省委、省政府和市委的坚强领导下，在市人大和市政协的监督支持下，坚定信心，凝聚智慧，克难攻坚，锐意进取，以更加振奋的精神、更加开阔的视野、更加务实的作风，投身于中原经济区建设的伟大实践，投身于周口富民强市的宏伟征程，为实现经济社会发展新跨越而努力奋斗！
